# Robot-assisted Radical Prostatectomy in the Brazilian Unified Health System (SUS): A Landmark in Surgical Public Health

**DOI:** 10.1590/S1677-5538.IBJU.2025.9918

**Published:** 2025-10-10

**Authors:** Leonardo O. Reis

**Affiliations:** 1 Universidade Estadual de Campinas Faculdade de Ciências Médicas Campinas SP Brasil UroScience, Faculdade de Ciências Médicas, Universidade Estadual de Campinas (UNICAMP), Campinas, SP, Brasil; 2 Pontifícia Universidade Católica de Campinas Campinas SP Brasil ImunOncologia, Pontifícia Universidade Católica de Campinas (PUC-Campinas), Campinas, SP, Brasil; 3 Instituto Nacional de Ciência, Tecnologia e Inovação em Câncer Geniturinário Campinas SP Brasil INCT UroGen, Instituto Nacional de Ciência, Tecnologia e Inovação em Câncer Geniturinário, Campinas, SP, Brasil

## COMMENT

The recent decision by the Brazilian Ministry of Health to incorporate robot-assisted radical prostatectomy (RARP) into the Brazilian Unified Health System (SUS) for clinically localized or locally advanced prostate cancer represents a landmark in the evolution of surgical care. For the first time, one of the world's most advanced and high-cost surgical technologies has been formally integrated into a universal public health system, positioning Brazil as a pioneer in equitable access to surgical innovation in Latin America.

Integrating high-cost technology such as RARP into a universal system like SUS extends far beyond clinical outcomes; it establishes surgical innovation as a driver of industrial policy, knowledge transfer, and local capacity building. Beyond representing technological progress, this decision underscores SUS's ability, the largest universal, tax-funded health system globally, to integrate cutting-edge surgical technologies while maintaining its principles of universality, equity, and integrality (1).

The National Commission for the Incorporation of Technologies into SUS (CONITEC) thoroughly evaluated clinical, economic, and societal evidence through public consultation No. 50/2025, which received 149 contributions from patient groups, professional associations, and civil society, with 99.3% supporting incorporation. This alignment with patient-centered values, emphasizing quality of life, culminated in the publication of Portaria SECTICS/MS No. 72/2025 (2).

Implementation, however, will not be immediate or uniform. Robotic platforms require substantial investment, specialized training, and sustainable maintenance. Effective integration will depend on regionalization and concentration of procedures in high-volume centers, where public–private partnerships will be essential. Portaria SECTICS/MS No. 72/2025 allows 180 days for operationalization, a timeline that will test federal, state, and institutional capacities. A hub-and-spoke model, concentrating RARP in accredited reference centers, may optimize resources while ensuring safety and quality.

This formal recognition transforms RARP from a technology previously confined to private institutions and select university hospitals into a standard of care progressively available across the world's largest universal health system. It exemplifies population-level health innovation, demonstrating that clinical excellence can coexist with social responsibility and that universal access can encompass high-complexity care (3, 4). Importantly, surgical innovation should be evaluated not only by technological sophistication but also by its impact on health outcomes, equity, and value. In this context, prospective registries, population-based studies, and health technology assessments are essential tools for measuring effectiveness, cost-effectiveness, and equity (3).

Brazil faces one of the highest burdens of prostate cancer globally, with over 70,000 new cases annually (5). While RARP has become the preferred surgical approach in high-income settings, SUS patients have historically relied on open or laparoscopic surgery. Incorporating RARP directly addresses this inequity, honoring the constitutional principle of universality that underpins Brazilian public health (1).

By integrating RARP into SUS, Brazil establishes a living laboratory for evaluating surgical care delivery across multiple dimensions: oncological outcomes (e.g., margin status, biochemical recurrence), functional recovery (continence, sexual function), perioperative complications, and continuous professional development. This approach ensures equitable access to high-complexity surgery, independent of socioeconomic status or geographic location, while enabling systematic assessment of cost-effectiveness and long-term societal impact, including return to productivity and reduction in morbidity. Importantly, this real-world implementation provides an unprecedented opportunity to challenge and refine the "Natural History" of evidence in radical prostatectomy, as recently examined through the "Reverse Systematic Review" strategy proposed by our UroScience/INCT UroGen team (4-7).

Mandatory national registries for RARP within SUS will generate one of the largest real-world evidence platforms worldwide, allowing continuous assessment of effectiveness, equity, and cost-effectiveness. Brazil can thus become a global leader in the science of surgical care delivery, addressing critical questions: Which patient subgroups benefit most? How do outcomes vary across regions and institutions? What is the system-level cost-effectiveness compared to traditional approaches? How can quality improvement optimize safety, functional recovery, and equity simultaneously (3)?

Integrating high-cost technology also creates opportunities to negotiate prices, foster domestic production, and stimulate knowledge transfer, reducing dependence on imported solutions. This aligns with global surgical public health research, which emphasizes studying real-world effectiveness, safety, and system-wide value rather than solely procedural efficacy (8). The timing is particularly strategic: core patents for robotic surgical systems expired between 2019 and 2022, enabling the entry of multiple new platforms into the market (Figure). Through SUS, Brazil's procurement power can be leveraged to negotiate competitive pricing, encourage local assembly, and promote research and workforce development, thereby strengthening the local Health Economic-Industrial Complex and advancing technological sovereignty.

**Figure:**
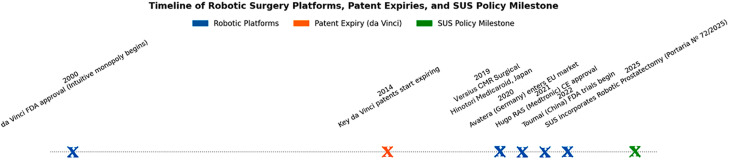
Timeline of Robotic Surgery Platforms, Patent Expiries, and SUS Policy Milestone (2000-2025).

- **2000–2014**: Intuitive monopoly with da Vinci.
- **2014 onward**: Patent expirations, market opening.
- **2019–2022**: Entry of competitors (Versius, Hinotori, Avatera, Hugo RAS, Toumai).
- **2025**: Strategic SUS incorporation of robotic prostatectomy (Portaria Nº 72/2025).

Brazil's decision positions SUS as a global reference in equitable surgical innovation, demonstrating that universal systems can offer cutting-edge care without compromising sustainability. While RARP involves higher upfront costs, medium- and long-term benefits, such as faster return to productivity and reduced functional morbidity, may support cost-effectiveness from a population health perspective (9).

Successful implementation requires addressing several critical challenges: training surgeons and multidisciplinary teams in robotic techniques; establishing surgical hubs equipped with operational platforms, maintenance capacity, and rigorous safety protocols; organizing equitable patient pathways; and implementing robust monitoring systems for outcomes, cost-effectiveness, and quality of care. Furthermore, hospital eligibility should be guided by strict adherence to clinical guidelines, competitive procurement practices, reliable local supply chains, accreditation standards, and minimum procedural volume thresholds to ensure both safety and optimal outcomes.

The incorporation of RARP into SUS represents more than a clinical advancement; it constitutes a strategic opportunity. By integrating clinical science, industrial policy, and public health principles, Brazil can simultaneously enhance patient outcomes and foster economic development, knowledge transfer, and technological independence. Implementing robust evaluation frameworks will be essential to assess not only surgical outcomes but also equity, quality, and long-term value. In doing so, SUS will continue to prioritize broad access over concentrated provision, ensuring the ethical, evidence-based, and sustainable use of healthcare resources.
